# Topographic and Surgical Risk Factors for Early Myopic Regression between Small Incision Lenticule Extraction and Laser In Situ Keratomileusis

**DOI:** 10.3390/diagnostics14121275

**Published:** 2024-06-17

**Authors:** Chia-Yi Lee, Yu-Ting Jeng, Shun-Fa Yang, Chin-Te Huang, Chen-Cheng Chao, Ie-Bin Lian, Jing-Yang Huang, Chao-Kai Chang

**Affiliations:** 1 Institute of Medicine, Chung Shan Medical University, Taichung 402, Taiwan; 2 Nobel Eye Institute, Taipei 115, Taiwan; 3 Department of Ophthalmology, Jen-Ai Hospital Dali Branch, Taichung 412, Taiwan; 4 Department of Medical Research, Chung Shan Medical University Hospital, Taichung 402, Taiwan; 5 Department of Ophthalmology, Chung Shan Medical University Hospital, Taichung 402, Taiwan; 6 Department of Ophthalmology, School of Medicine, Chung Shan Medical University, Taichung 402, Taiwan; 7 Department of Optometry, MacKay Junior College of Medicine, Nursing, and Management, Taipei 112, Taiwan; 8 Institute of Statistical and Information Science, National Changhua University of Education, Changhua 500, Taiwan; 9 Department of Optometry, Da-Yeh University, Chunghua 515, Taiwan

**Keywords:** small-incision lenticule extraction, laser in situ keratomileusis, myopic regression, corneal curvature, central corneal thickness

## Abstract

Our objective was to evaluate the topographic and surgical factors of early myopic regression between laser in situ keratomileusis (LASIK) and small-incision lenticule extraction (SMILE). A retrospective case–control study was conducted, and 368 and 92 eyes were enrolled in the LASIK and SMILE groups via propensity score matching (PSM). Visual acuity, refractive status, axial length, and topographic/surgical parameters were collected. Multiple linear regression was applied to the yield coefficient and the 95% confidence interval (CI) of the parameters. The cumulative incidence of early myopic regression was higher in the LASIK group (*p* < 0.001). In the SMILE group, a lower central corneal thickness (CCT) thinnest value and a higher corneal cylinder associated with early myopic regression were observed; meanwhile, in the LASIK group, a lower CCT thinnest value, a higher steep corneal curvature, a larger optic zone, and a lower flap thickness related to early myopic regression were observed (all *p* < 0.05). In the SMILE group, a higher CCT difference correlated with early myopic regression was observed compared to the LASIK group (*p* = 0.030), and higher steep corneal curvature and lower cap/flap thickness (both *p* < 0.05) correlated with early myopic regression were observed in the LASIK group compared to the SMILE group. In conclusion, CCT differences significantly influence early myopic regression in the SMILE group; meanwhile, corneal curvature and flap thickness affect early myopic regression principally in the LASIK group.

## 1. Introduction

Over the last 20 to 30 years, corneal refractive surgeries have been introduced for the correction of myopia [1]. In relation to the type of corneal refractive surgery, photorefractive keratectomy, laser in situ keratomileusis (LASIK), and small-incision lenticule extraction (SMILE) are widely applied techniques [2]. In the past decades, the number of patients receiving SMILE surgery has increased, which may result from the ensuing improved comfort and lower dryness [3]. In a comparison between the LASIK and SMILE surgeries, the efficiency and predictability of LASIK and SMILE showed no prominent differences [4,5], while the SMILE surgery demonstrated better postoperative corneal sensitivity in a previous publication [6]. However, the optical density that could result in blurry vision was higher in individuals who received the SMILE procedure than in individuals who received LASIK surgery within one month postoperatively [7].

Although the corneal refractive surgeries showed reasonable levels of safety, some postoperative complications can occur after either LASIK or SMILE surgeries [8]. The common postoperative complications of SMILE and LASIK surgeries include dry eye disease, superficial keratitis, diffuse lamellar keratitis, epithelial ingrowth, infectious keratitis, and postoperative corneal ectasia [9,10]. Moreover, myopic regression is both the natural course and a not-uncommon postoperative complication of LASIK and SMILE procedures [11,12,13]. Natural myopic regression after LASIK or SMILE surgeries does not lead to significant visual impairment [14,15], while early myopic regression developed over months can cause visual disturbance [16]. According to previous research, it has been estimated that between 4 and 20 percent of SMILE and LASIK individuals developed early myopic progression, so an enhancement procedure was required for them [10,17]. As a result, it is important to identify potential risk factors for the early myopic regression of corneal refractive surgeries.

The preoperative risk factors for early myopic regression in LASIK surgery have been identified, among which a high degree of myopia, a large optic zone, and female gender were associated with a higher possibility of early myopic regression in LASIK surgery [11,18]. However, the topographic risk factors for early myopic progression after SMILE surgery have seldom been reported. Furthermore, the surgical methods of the LASIK and SMILE procedures are different [2], and so whether the predisposing factors of early myopic regression between the two surgeries are different requires further validation.

Consequently, the purpose of our study is to investigate the difference in topographic and surgical risk factors for early myopic regression between SMILE and LASIK surgeries. In addition, the risk factors for early myopic regression for SMILE and LASIK surgeries were analyzed.

## 2. Materials and Methods

### 2.1. Participant Selection

A retrospective case–control study was conducted at the Nobel Eye Clinic, Kaohsiung branch, which is a local clinic that specializes in cataract and refractive surgeries in Southern Taiwan. The individuals enrolled in our study met the following criteria: (1) age between 18 and 55 years; (2) received SMILE or LASIK surgery at the Kaohsiung Nobel Eye Clinic; and (3) the existence of myopia of at least −1.00 diopter (D). In general, the patient would select the surgery they wanted to receive after thorough discussion with the physician and realizing the advantages and disadvantages of both LASIK and SMILE surgeries. However, in certain situations, the clinical team arranged LASIK, which included (1) a sphere power of more than −10.00 D, (2) a cylinder power of more than −5.00 D, and (3) an estimated RST lower than 280μm in the SMILE calculation program. Moreover, the following exclusion criteria were adopted to exclude individuals with significant ocular diseases: (1) an uncorrected visual acuity (UCVA) of less than 20/400 on the Snellen chart, (2) a diagnosis of cataracts, (3) a diagnosis of any form of keratoconus or other corneal ectatic diseases, (4) a diagnosis of severe dry eye disease, central corneal opacity, corneal neovascularization, and active recurrent corneal erosion, (5) a diagnosis of retinal disorder involving vitreous hemorrhage, proliferative diabetic retinopathy, macular pucker, and retinal detachment, (6) a diagnosis of unstable or uncontrolled glaucoma, (7) a diagnosis of eyeball rupture and severe ptosis, (8) a diagnosis of optic neuropathy, (9) unstable refractive status in which the myopia progressed more than 0.5 D within two years, (10) pregnancy, and (11) active systemic inflammatory diseases including diabetes mellitus, thyroid disease, systemic lupus erythematous, Sjögren syndrome, ankylosing spondylitis, rheumatic arthritis, and systemic sclerosis. Then, eyes that received LASIK surgery were matched to four eyes that received SMILE surgery via the propensity score matching (PSM) method. The PSM incorporated age, sex, BCVA, preoperative cycloplegic refraction, AXL, and the topographic indexes between the two groups. Finally, a total of 368 and 92 eyes were categorized into the SMILE group and the LASIK group, respectively.

### 2.2. Surgical Technique

All SMILE and LASIK surgeries in our study were performed by two experienced refractive specialists (Y.-T.J. and C.-K.C.). The SMILE procedure was completed by a femtosecond laser device (Visuamax 500; Carl Zeiss, Göschwitzer Str., Jena, Germany). The optic zone was set to 5.5–6.9 mm based on the lenticular thickness and pupil size, and the corneal incision was set to 3.0 mm and created at 105 degrees. After the angle kappa was confirmed with a microscope using the coaxial-sighted corneal light reflex method, the cornea was fixed using a suction ring. Then, the femtosecond laser was triggered. After the femtosecond laser emission, a spatula was used to dissect the upper and lower interfaces of the corneal lenticule, and then the corneal lenticule was extracted using forceps. The LASIK surgery was performed using a femtosecond device (Visuamax 500; Carl Zeiss, Göschwitzer Str., Jena, Germany) and an excimer laser device (EX500; Alcon, Fort Worth, TX, USA). The excimer laser device in our institution applied the wavefront ablation profile. The corneal flap was created with the femtosecond device, with a flap diameter ranging from 6.5 to 9.0, depending on the ablation depth and pupil size. Then, the corneal flap was elevated with a spatula, and the pupil center was defined by the surgeon. After that, the excimer laser was emitted, and a prednisolone suspension was instilled into the stromal bed after completion of the laser strike. The corneal flap and stromal bed were irrigated using normal saline, and the flap was placed back onto the stromal bed. Following that, a soft contact lens was placed on the corneal surface for one day. After the surgery, levofloxacin eye drops and prednisolone eye drops were applied for about one week; then, the eyedrops were changed to sulfamethoxazole and fluorometholone eye drops for another three weeks. The nomograms for myopia compensation in the SMILE and LASIK surgeries are available in Supplementary Table S1.

### 2.3. Ophthalmic Examination

All participants who received the SMILE and LASIK procedures received identical ophthalmic examinations at the Nobel Eye Clinic, Kaohsiung branch. The data before refractive surgery, one week after refractive surgery, one month after refractive surgery, three months after refractive surgery, and six months after refractive surgery were recorded. All the patients completed the six-month follow-up. The preoperative exams included manifest refraction with best-corrected visual acuity (BCVA) and cycloplegic refraction of sphere and cylinder powers by autorefractor (KR-8900; Topcon, Itabashi-ku, Tokyo, Japan); central corneal thickness (CCT) with corneal apex and thinnest evaluation, steep and flat corneal curvature of anterior as well as posterior corneal surface, and corneal cylinder power of anterior as well as posterior corneal surface using a topographic machine (Oculus Pentacam; OCULUS Optikgeräte GmbH, Münchholzhäuser, Wetzlar, Germany); and axial length (AXL) using a biometry machine (IOL Master 500; Carl Zeiss, Göschwitzer Str., Jena, Germany). The postoperative examinations of our patients included UCVA, BCVA, manifest sphere power and cylinder power, CCT, corneal curvature, corneal cylinder power, and AXL using identical devices. Moreover, the surgical parameters, including optic zone, cap/flap thickness, residual stromal thickness (RST), lenticular thickness of SMILE surgery, and laser ablation depth of LASIK surgery, were obtained. The spherical equivalent was defined as sphere power plus half the cylinder power, and the difference in CCT was defined as the CCT of the apex minus the CCT of the thinnest area in our study. In addition, early myopic regression referred to a myopic shift of more than −0.75 D during the 6-month follow-up period in our study. We defined myopic regression as a myopic shift of more than −0.75 D after considering the previous definitions of myopic regression in the literature [12,19]. In addition, non-regressive population was defined as those without a myopic shift or with a myopic shift lower than −0.75 D.

### 2.4. Statistical Analysis

SPSS version 20.0 (SPSS Inc., Chicago, IL, USA) was utilized for the statistical analyses in our study. The Shapiro–Wilk test was executed to confirm whether the data of the SMILE and LASIK groups presented a normal distribution, which revealed normal distribution statuses in all whole-group data (all *p* > 0.05). Still, the data from the early myopic regression populations did not present a normal distribution (all *p* < 0.05). The sample size calculation was based on statistical power, and the statistical power of our study was 0.95 with a 0.05 alpha value and a medium effect size, which was generated using the G*power version 3.1.9.2 (Heinrich Heine Universität at Düsseldorf, Germany). The descriptive analysis was employed to illustrate age, sex, refraction status, topographic characters, AXL, and surgical parameters; then, an independent T test and Fisher’s exact test were used to evaluate the values of the above indexes between the two groups. The independent T test was also adopted to present the efficiency and predictability between the SMILE and LASIK groups six months after surgery. Fisher’s exact test and Mann–Whitney U test were executed to compare the preoperative and postoperative characters between the regression populations in the SMILE and LASIK groups. For the percentage of early myopic regression and the difference in regression episode between groups, the survival analysis with a Kaplan–Meier curve was executed. For the risk factors of early myopic regression of SMILE and LASIK surgeries, we applied a multiple linear regression for preoperative refraction, topographic factors, and surgical parameters with adjustments for age and sex, and the coefficient with a 95% confidence interval (CI) of each potential risk factor was presented. We chose age and sex as parameters in the regression model because they represent basic demographic data. Concerning the other parameters in the regression model, we selected preoperative refraction, topographic factors, and surgical parameters because they are vital to the efficiency and predictability of refractive surgery, according to our experience. In addition, many research studies have included these factors for analyzing myopic regression after refractive surgery [11,12,18,20]. In the next step, the interaction test was utilized to analyze the prominent risk-protective factors of early myopic regression in SMILE surgery compared to LASIK surgery based on the results of multiple linear regression. A *p* value < 0.05 was set as statistically significant, and a *p* value lower than 0.001 was presented as *p* < 0.001 in our study.

## 3. Results

The baseline characteristics of the SMILE and LASIK groups are presented in Table 1. The mean age was 31.21 ± 4.23 and 31.09 ± 5.02 years old in the SMILE and LASIK groups, respectively, without a significant difference (*p* = 0.833). The sex distribution, BCVA, and refraction status also showed no significant difference between the two groups (all *p* > 0.05). In relation to the topographic and surgical parameters, the LASIK group showed a thicker RST compared to the SMILE group (*p* = 0.002), while other indexes showed similar values between the two groups (all *p* > 0.05) (Table 1).

**Figure 1:**
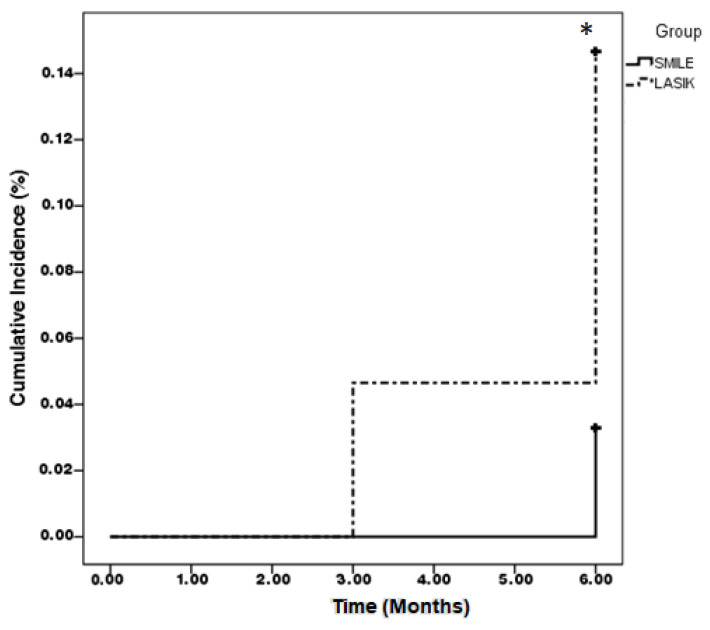
Kaplan–Meier curve of early myopic regression between the two groups. * denotes a significant difference between the two groups (*p* < 0.001).

After the 6-month follow-up period, the UCVA was significantly better in the SMILE group compared to the LASIK group (*p* < 0.001). The manifest SE and sphere power showed a significant difference between the two groups (both *p* < 0.05), and the LASIK group demonstrated a large CCT difference and lower corneal cylinder power compared to the SMILE group (all *p* < 0.05) (Table 2). There were 12 (3.26%) and 13 (14.13%) patients who manifested an early myopic regression within 6 months after surgery in the SMILE and LASIK groups, respectively, and their preoperative and postoperative metrics are presented in Table 3. The survival analyses showed a significantly higher cumulative incidence of early myopic regression in the LASIK group than the SMILE group (*p* < 0.001) (Figure 1).

Concerning the risk factors of early myopic regression in the SMILE surgery, a lower CCT thinnest value and a higher corneal cylinder were associated with the development of early myopic regression (both *p* < 0.05) (Table 4). For the LASIK surgery, a lower CCT thinnest value, a higher steep corneal curvature, a larger optic zone, and a lower flap thickness were related to the existence of early myopic regression (all *p* < 0.05) (Table 5). In addition, in the SMILE group, a higher CCT difference was correlated with a higher chance of early myopic regression compared with the LASIK group (*p* = 0.030). In the LASIK group, higher steep corneal curvature (*p* = 0.003) and lower cap/flap thickness (*p* = 0.004) were correlated with a higher possibility of early myopic regression compared to the SMILE group (Table 6).

## 4. Discussion

In our study, the LASIK surgery presented a higher incidence of early myopic regression compared to the SMILE surgery. Moreover, the CCT difference influenced early myopic regression more significantly in the SMILE surgery than in the LASIK surgery, while steep corneal curvature and flap thickness were related to early myopic regression mainly in the LASIK surgery rather than in the SMILE surgery. In addition, in the SMILE surgery, the topographic factor was correlated with early myopic regression, and both the topographic and surgical factors were associated with early myopic regression in the LASIK surgery.

Certain hypotheses about early myopic regression after corneal refractive surgeries have been proposed [18]. In a previous study, the manifestation of corneal epithelium thickening could be a possible mechanism of early myopic regression following LASIK surgery [16]. The CCT, which includes the epithelial and stromal thicknesses, increased significantly three months after the LASIK surgery in individuals with early myopic regression [21]. It is possible that the laser ablation may induce the wound-healing process of the cornea and thereby contribute to corneal epithelium proliferation [22]. Additionally, the existence of postoperative dry eye following the LASIK surgery could impair the ocular surface and change the corneal epithelium structure [23]. In relation to the molecular pathway, corneal epithelium proliferation was observed under high-level inflammatory cytokine expression such as growth factors and insulin-like growth factor-1 [24,25], which were present in the cornea after the LASIK surgery [26]. In addition to epithelial proliferation, the forward shift of the corneal curvature could be another cause of early myopic regression in corneal refractive surgery [16]. The corneal strength after corneal refractive surgeries like photorefractive keratectomy, LASIK, and SMILE tended to decrease, and the SMILE surgery demonstrated the highest residual corneal stiffness [27]. The weakened corneal structure could cause the anterior movement of the corneal curvature and the subsequent myopic regression [28,29]. This hypothesis was further supported by the increment in total corneal refractive power after corneal refractive surgery, despite there being no dominant myopic regression identified in previous research [30]. On the other hand, myopic formation can result from axial elongation or corneal curvature steepening [31], and whether the postoperative axial elongation existed during the formation of early myopic regression has not yet been fully elucidated. According to our results, the AXL in both the SMILE and LASIK groups did not increase after the surgery; thus, early myopic regression may not result from AXL elongation. Moreover, several topographic factors, including the CCT at the thinnest part, the corneal cylinder power, and the steep corneal curvature, were correlated with the development of early myopic regression in either the SMILE group or the LASIK group. The above findings in our study may further prove that the causation of early myopic regression in both the SMILE and LASIK surgeries was attributable to the alteration in corneal structure after surgery.

Early myopic regression after the LASIK surgery was associated with a lower CCT thinnest part, a higher steep corneal curvature, a larger optic zone, and a lower flap thickness. In previous studies in the literature, a higher corneal curvature and a large optic zone were both associated with the development of early myopic regression in patients who underwent the LASIK procedure, and our findings further confirmed their correlation with myopic regression [11,18,32]. Thin CCT was recognized as a predisposing factor for the development of postoperative corneal ectasia and irregular astigmatism in a previous publication [33]. Because of the careful selection of patients, no prominent corneal ectasia was observed in our study population during this study. However, the thin cornea at a specific site may relate to the forward movement of the cornea after refractive surgery and following a myopic shift. The possible reason for the association between lower flap thickness and early myopic regression in the LASIK surgery may be due to the disruption of Bowman’s layer and the anterior stroma in the thin flap, which is a region that maintains the corneal shape [34]. In addition, the reduction in flap thickness for a thin cornea scheduled for LASIK surgery is common; meanwhile, a thin cornea itself is correlated with corneal ectasia [33]. In relation to the SMILE surgery, high myopia and astigmatism were shown to be potential risk factors for undercorrection and enhancement after SMILE surgery in a previous study [35]. In our study, a lower CCT at its thinnest part and a higher corneal cylinder were correlated with early myopic regression. The lower CCT at its thinnest part is also a risk factor for early myopic regression in the LASIK surgery, according to the findings of our study. Thus, the anterior shift from the thinnest part of the cornea might have also resulted from the myopic formation in the SMILE surgery. A higher corneal cylinder is another risk factor for early myopic regression after SMILE surgery, which is reasonable since high astigmatism could influence the corneal structure and precision of the surgery [35]. Of note, the correlation between CCT difference and early myopic regression in SMILE surgery was marginally significant, and a significant correlation between them may have been shown with more cases; however, further research is required to prove this hypothesis.

Concerning the difference in each parameter on the development of early myopic regression in different refractive surgeries, a high CCT difference was related to a higher risk of early myopic regression in the SMILE surgery, while a high steep corneal curvature and low cap/flap thickness were related to a higher chance of early myopic regression in the LASIK surgery. In a previous study, the existence of significant myopia was a risk factor for early myopic regression in both the LASIK and SMILE surgeries [11,18,35]. However, there were no available studies in the literature that compared the different effects of ophthalmic parameters on early myopic regression in each refractive surgery. To our knowledge, our revelation of the ophthalmic parameters that show different effects on myopic regression between the LASIK and SMILE surgeries may be the first of such studies. In addition, age and sex, being risk factors for early myopic regression of LASIK [11,18], were adjusted in the multiple logistic regression model; thus, our results may possess reasonable significance. In addition, the same devices were employed for all the refractive surgeries in our study, and the procedures were performed by two experienced surgeons; therefore, the homogeneity of the surgery itself may be adequate. Although the factors for early myopic regression in different refractive surgeries were displayed [12,20], some parameters analyzed in our study, such as the CCT difference and the AXL, were not evaluated in other studies. Moreover, we identified certain factors that were only related to myopic regression in SMILE surgery but not in LASIK surgery (i.e., a higher CCT difference) or in LASIK surgery but not in SMILE surgery (i.e., a higher steep corneal curvature and lower cap/flap thickness) for nearly all degrees of myopia, and these were not reported in previous research studies [12,20]. In the analysis of each group, a higher CCT difference between the apex and thinnest part demonstrated a marginally positive effect on early myopic regression in the SMILE group but a non-prominent effect in the LASIK group. We speculate that the high difference in CCT may contribute to a more irregular interface in the SMILE surgery than the LASIK surgery since the LASIK flap would be attached to the RST at the end of surgery, and the chance of an anterior shift of the residual stromal bed would therefore be lower in the LASIK surgery. On the other hand, in the LASIK surgery group, a higher steep corneal curvature and lower cap/flap thickness were more significantly correlated with early myopic regression compared to the SMILE surgery group. The anterior forward effect of the cornea is one of the mechanisms of early myopic regression following refractive surgeries [16], and the biomechanical strength of the cornea was lower in the LASIK surgery than in the SMILE surgery [27]. Consequently, the steeper corneal curvature and the thin cap/flap that disturbed the firm Bowman’s layer and the anterior stroma may contribute to a more changeable corneal structure and subsequent anterior corneal displacement in the LASIK surgery compared to the SMILE surgery.

Concerning the treatment efficiency and predictability of refractive surgeries in our study, the mean postoperative UCVA was 0.98 ± 0.06 and 0.99 ± 0.14 one month and six months postoperatively in the SMILE group, and 96% of individuals who received SMILE surgery achieved a UCVA of 20/25 or better one month postoperatively. Previous research illustrated that between 82 and 96 percent of patients achieved a UCVA better than 20/25 three months after SMILE surgery; therefore, the efficiency of our study is compatible with earlier experiences [36,37]. For predictability, the SE was −0.03 D one month postoperatively in the SMILE group in our study, which was also similar to the findings in previous publications that demonstrated a postoperative SE from −0.28 D to −0.38 D [37,38]. On the other hand, the mean postoperative UCVA was 0.97 ± 0.11 and 0.86 ± 0.32 one month and six months postoperatively in the LASIK group, and 92% of individuals who received LASIK surgery achieved a UCVA of 20/25 or better one month postoperatively. In previous publications discussing LASIK surgery, the percentage that reached a UCVA of 20/25 or better after one month was from 80 to 100 percent [36,39,40]. The postoperative mean SE was −0.17 D in our LASIK group, which was also within the −0.02 D to −0.19 D range from previous articles [41,42]. In our study, the rate of early myopic regression was about four-fold higher in the LASIK group compared to the SMILE group, which is similar to the regression ratio between the SMILE and LASIK surgeries presented in earlier articles [10,17].

There were several limitations to our study. Firstly, the retrospective nature of our study design decreased the homogeneity of the study population even after the PSM process. Secondly, the patient numbers between the SMILE and LASIK groups were imbalanced, as the SMILE group had four times more participants than the LASIK group because we wanted to present as many cases as possible. In addition, some parameters of corneal structure, including the Belin–Ambrosio enhanced ectasia total deviation index and the tomographic biomechanical index, were not available in our study due to the absence of related devices. Moreover, the epithelial thickness value was absent since we did not perform related tests in routine examinations, and the lack of postoperative cycloplegic refraction was another prominent limitation because we did not arrange it as a routine postoperative exam. Finally, we did not perform cycloplegic refraction after the refractive surgery; therefore, the refractive status of our participants may not be completely accurate.

## 5. Conclusions

In conclusion, the CCT difference caused a higher chance of early myopic regression in the SMILE surgery, while the higher steep corneal curvature and lower cap/flap thickness led to a higher possibility of early myopic regression in the LASIK surgery. Furthermore, the CCT at its thinnest part correlated with early myopic regression in both the SMILE and LASIK surgeries. Consequently, the above risk factors might be considered while choosing the type of refractive surgery to decrease the rate of early myopic regression. Further prospective, large-scale research to evaluate the risk factors of overcorrection in each refractive surgery is mandatory.

## Figures and Tables

**Table 1:** Basic features of the study population.

Characteristics	SMILE Group (N = 368)	LASIK Group (N = 92)	*p*
Age (years)	31.21 ± 4.23	31.09 ± 5.02	0.833
Sex	160:208	41:51	0.390
BCVA	0.99 ± 0.00	0.99 ± 0.01	0.988
Manifest refraction (D)			
SE	−5.72 ± 2.04	−5.69 ± 2.47	0.914
Sphere	−5.23 ± 1.86	−5.17 ± 2.20	0.810
Cylinder	−0.98 ± 0.74	−1.04 ± 0.80	0.515
Cycloplegic refraction (D)			
SE	−5.88 ± 2.08	−5.86 ± 2.57	0.945
Sphere	−5.34 ± 1.97	−5.28 ± 2.42	0.826
Cylinder	−1.08 ± 0.81	−1.16 ± 0.93	0.451
AXL (mm)	25.97 ± 1.02	25.88 ± 1.20	0.509
CCT (μm)			
Apex	550.13 ± 29.83	552.67 ± 26.11	0.419
Thinnest	545.29 ± 30.35	548.14 ± 26.72	0.375
Difference	4.84 ± 2.27	4.53 ± 3.48	0.418
Anterior corneal curvature (D)			
Steep corneal curvature	44.21 ± 1.19	44.38 ± 1.28	0.250
Flat corneal curvature	42.78 ± 1.15	42.90 ± 1.29	0.417
Corneal cylinder	−1.43 ± 0.71	−1.48 ± 0.76	0.569
Posterior corneal curvature (D)			
Steep corneal curvature	−6.33 ± 0.15	−6.21 ± 0.24	0.265
Flat corneal curvature	−5.84 ± 0.14	−5.86 ± 0.11	0.803
Corneal cylinder	−0.48 ± 0.04	−0.35 ± 0.19	0.190
Optic zone (mm)	6.38 ± 0.37	6.29 ± 0.40	0.052
Cap thickness (μm)	107.13 ± 8.42	106.22 ± 4.77	0.171
RST (μm)	326.61 ± 30.68	343.45 ± 39.16	0.002 *
Laser depth (μm)	110.16 ± 27.03	103.42 ± 30.28	0.053

AXL: axial length; BCVA: best-corrected visual acuity; CCT: central corneal thickness; D: diopter; LASIK: laser in situ keratomileusis; N: number; RST: residual stromal thickness; SE: spherical equivalent; SMILE: small-incision lenticule extraction. * denotes a significant difference between groups.

**Table 2:** Six-month postoperative outcomes between the SMILE and LASIK groups.

Index	SMILE Group (N = 368)	LASIK Group (N = 92)	*p* Value
UCVA	0.99 ± 0.14	0.86 ± 0.32	<0.001 *
BCVA	0.99 ± 0.01	0.97 ± 0.20	0.340
Manifest refraction (D)			
SE	−0.04 ± 0.11	−0.17 ± 0.09	<0.001 *
Sphere	−0.02 ± 0.15	−0.16 ± 0.12	<0.001 *
Cylinder	−0.03 ± 0.08	−0.03 ± 0.11	0.998
AXL (mm)	25.59 ± 2.52	25.58 ± 2.93	0.990
CCT (μm)			
Apex	447.57 ± 53.69	456.94 ± 46.32	0.125
Thinnest	445.61 ± 52.57	454.11 ± 55.78	0.171
Difference	1.96 ± 1.53	2.83 ± 1.47	<0.001 *
Anterior corneal curvature (D)			
Steep corneal curvature	38.84 ± 4.06	39.30 ± 4.82	0.200
Flat corneal curvature	38.05 ± 3.98	38.66 ± 5.56	0.162
Corneal cylinder	−0.79 ± 0.41	−0.64 ± 0.48	0.007 *
Posterior corneal curvature (D)			
Steep corneal curvature	−6.33 ± 0.15	−6.21 ± 0.23	0.296
Flat corneal curvature	−5.83 ± 0.16	−5.82 ± 0.05	0.948
Corneal cylinder	−0.50 ± 0.08	−0.37 ± 0.21	0.118

AXL: axial length; BCVA: best-corrected visual acuity; CCT: central corneal thickness; D: diopter; LASIK: laser in situ keratomileusis; N: number; SE: spherical equivalent; SMILE: small-incision lenticule extraction; UCVA: uncorrected visual acuity. * denotes a significant difference between groups.

**Table 3:** Preoperative characteristics and postoperative outcomes in the regression population of SMILE and LASIK surgeries.

Characteristics	SMILE Group (N = 12)	LASIK Group (N = 13)	*p*
**Preoperative status**			
Age (year)	31.07 ± 4.93	30.36 ± 2.76	0.137
Sex	4:8	6:7	0.352
BCVA	0.99 ± 0.01	0.99 ± 0.01	0.998
Manifest refraction (D)			
SE	−5.66 ± 2.70	−5.79 ± 2.49	0.477
Sphere	−4.85 ± 2.88	−5.20 ± 2.18	0.345
Cylinder	−1.63 ± 1.37	−1.20 ± 0.77	0.135
Cycloplegic refraction (D)			
SE	−5.43 ± 2.99	5.60 ± 2.65	0.794
Sphere	−4.60 ± 3.16	−4.95 ± 2.34	0.544
Cylinder	−1.66 ± 1.44	−1.30 ± 0.86	0.701
AXL (mm)	25.60 ± 1.61	25.77 ± 0.83	0.874
CCT (μm)			
Apex	546.36 ± 27.71	548.37 ± 25.12	0.185
Thinnest	541.43 ± 27.20	544.21 ± 26.36	0.294
Difference	4.93 ± 1.82	4.14 ± 2.18	0.069
Anterior corneal curvature (D)			
Steep corneal curvature	44.60 ± 1.28	45.09 ± 1.58	0.401
Flat corneal curvature	42.93 ± 0.68	43.64 ± 1.23	0.114
Corneal cylinder	−1.67 ± 1.33	−1.45 ± 0.53	0.439
Posterior corneal curvature (D)			
Steep corneal curvature	−6.43 ± 0.17	−6.24 ± 0.29	0.187
Flat corneal curvature	−5.93 ± 0.15	−5.85 ± 0.17	0.679
Corneal cylinder	0.50 ± 0.07	0.39 ± 0.21	0.206
Optic zone (mm)	6.34 ± 0.31	6.37 ± 0.47	0.307
Cap/flap thickness (μm)	107.14 ± 9.94	102.57 ± 4.97	0.571
RST (μm)	323.50 ± 30.59	338.71 ± 30.88	0.329
Laser depth (μm)	110.29 ± 36.45	102.30 ± 26.93	0.125
**Postoperative status**			
UCVA	0.89 ± 0.07	0.83 ± 0.14	0.044 *
BCVA	0.98 ± 0.02	0.95 ± 0.07	0.281
Manifest refraction (D)			
SE	−1.32 ± 0.15	−1.37 ± 0.20	0.541
Sphere	−1.03 ± 0.12	−1.11 ± 0.17	0.497
Cylinder	0.58 ± 0.15	0.52 ± 0.18	0.798
AXL (mm)	25.60 ± 1.78	25.62 ± 2.22	0.494
CCT (μm)			
Apex	447.14 ± 33.99	451.93 ± 20.60	0.369
Thinnest	444.93 ± 33.07	449.36 ± 19.94	0.097
Difference	2.21 ± 1.25	2.57 ± 1.87	0.734
Anterior corneal curvature (D)			
Steep corneal curvature	38.15 ± 1.44	38.63 ± 1.39	0.727
Flat corneal curvature	37.32 ± 1.30	37.87 ± 1.39	0.654
Corneal cylinder	−0.75 ± 0.36	−0.74 ± 0.35	0.804
Posterior corneal curvature (D)			
Steep corneal curvature	−6.35 ± 0.11	−6.25 ± 0.20	0.417
Flat corneal curvature	−5.80 ± 0.14	−5.84 ± 0.05	0.245
Corneal cylinder	−0.55 ± 0.10	−0.41 ± 0.17	0.095

AXL: axial length; BCVA: best-corrected visual acuity; CCT: central corneal thickness; D: diopter; LASIK: laser in situ keratomileusis; N: number; RST: residual stromal thickness; SE: spherical equivalent; SMILE: small-incision lenticule extraction; UCVA: uncorrected visual acuity. * denotes a significant difference between groups.

**Table 4:** Preoperative risk factors for early myopic regression for SMILE surgery.

Factor	B	95% CI		*p* Value
		Lower Limit	Upper Limit	
**Cycloplegic refraction**				
Sphere	−0.186	−0.264	0.058	0.831
Cylinder	0.145	−0.068	0.187	0.812
AXL	0.116	−0.522	0.639	0.671
CCT				
Apex	0.081	−0.005	0.114	0.135
Thinnest	−0.666	−1.344	−0.038	0.010 *
Difference	0.677	−0.015	1.013	0.084
Anterior corneal curvature				
Steep corneal curvature	0.580	−0.057	1.193	0.599
Flat corneal curvature	−0.247	−0.387	0.264	0.827
Corneal cylinder	1.171	0.016	2.008	0.016 *
Posterior corneal curvature				
Steep corneal curvature	−0.137	−0.215	0.336	0.538
Flat corneal curvature	0.054	−0.116	0.178	0.945
Corneal cylinder	0.867	−0.224	1.213	0.354
Optic zone	0.742	−0.177	1.418	0.421
Cap thickness	−0.583	−0.714	0.095	0.112
RST	−0.742	−0.992	0.016	0.183
Lenticular thickness	−0.259	−0.923	0.587	0.295

AXL: axial length; CCT: central corneal thickness; CI: confidence interval; RST: residual stromal thickness. * denotes a significant difference between groups.

**Table 5:** Preoperative risk factors for early myopic regression for LASIK surgery.

Factor	B	95% CI		*p* Value
		Lower Limit	Upper Limit	
**Cycloplegic refraction**				
Sphere	0.075	−0.200	0.238	0.867
Cylinder	−0.429	−0.584	0.188	0.672
AXL	−1.050	−2.021	0.157	0.291
CCT				
Apex	0.482	−0.011	1.002	0.153
Thinnest	−0.530	−1.001	−0.002	0.048 *
Difference	−0.423	−1.000	0.118	0.688
Anterior corneal curvature				
Steep corneal curvature	1.614	0.073	2.389	0.001 *
Flat corneal curvature	−0.326	−1.078	0.051	0.690
Corneal cylinder	0.762	−0.072	1.167	0.504
Posterior corneal curvature				
Steep corneal curvature	−1.017	−1.944	1.463	0.225
Flat corneal curvature	0.297	−0.826	0.125	0.314
Corneal cylinder	0.102	−0.298	0.235	0.649
Optic zone	0.273	0.172	0.389	0.016 *
Flap thickness	−2.001	−3.131	−0.002	0.040 *
RST	−1.638	−1.991	0.009	0.089
Laser ablation depth	−0.511	−1.130	0.008	0.063

AXL: axial length; CCT: central corneal thickness; CI: confidence interval; RST: residual stromal thickness. * denotes a significant difference between groups.

**Table 6:** Preoperative risk factors for early myopic regression of SMILE surgery compared to LASIK surgery.

Factor	B	95% CI		*p* Value
		Lower Limit	Upper Limit	
**Cycloplegic refraction**				
Sphere	0.011	−0.645	0.811	0.992
Cylinder	0.033	−0.209	1.010	0.415
AXL	0.196	−0.017	0.270	0.082
CCT				
Apex	0.046	−0.097	0.095	0.993
Thinnest	−0.029	−0.126	0.081	0.238
Difference	0.098	0.006	0.140	0.033 *
Anterior corneal curvature				
Steep corneal curvature	−0.504	−1.841	−0.055	0.002 *
Flat corneal curvature	−0.003	−0.316	0.057	0.125
Corneal cylinder	0.057	−0.028	0.075	0.102
Posterior corneal curvature				
Steep corneal curvature	−0.206	−0.624	0.089	0.094
Flat corneal curvature	0.009	−0.411	0.238	0.543
Corneal cylinder	0.029	−0.018	0.040	0.538
Optic zone	−0.017	−0.115	0.069	0.198
Cap/flap thickness	−0.371	−1.232	−0.020	0.006 *
RST	0.194	−0.018	0.796	0.060
Lenticular thickness/Laser depth	−0.021	−0.674	0.111	0.339

AXL: axial length; CCT: central corneal thickness; CI: confidence interval; RST: residual stromal thickness. * denotes a significant difference between groups.

## Data Availability

The data applied in this study are available from the corresponding author upon reasonable request.

## Supplementary Materials

The following supporting information can be downloaded at https://www.mdpi.com/article/10.3390/diagnostics14121275/s1: Table S1: The nomogram of refractive surgery in this study.
