# Supplementary material for: Topographic and Surgical Risk Factors for Early Myopic Regression between Small Incision Lenticule Extraction and Laser In Situ Keratomileusis
Source: Diagnostics (Basel). 2024 Jun 17;14(12):1275. doi: 10.3390/diagnostics14121275 (PMC11202549; doi:10.3390/diagnostics14121275)
Supplement: Supplementary file 1 [file diagnostics-14-01275-s001.zip › diagnostics-3024690-supplementary.pdf]

Table S1. The nomogram of refractive surgery in this study.

**Myopia degree**	**SMILE**	**LASIK**
Lower than -2.50D	Target refraction	Target refraction
-2.50D to -5.00D	(Target refraction plus -0.15D)*1.1	Target refraction plus -0.15D
-5.00D to -7.50D	(Target refraction plus -0.25D)*1.1	Target refraction plus -0.25D
More than -7.50D	(Target refraction plus -0.40D)*1.1	Target refraction plus -0.40D

D: diopter; LASIK: laser in situ keratomileusis; SMILE: small incision lenticule extraction
